# 11β-Hydroxysteroid dehydrogenase type 1 deficiency causes sexual dimorphism in body composition and bone mass in response to caloric restriction

**DOI:** 10.1093/jbmrpl/ziag075

**Published:** 2026-04-17

**Authors:** Iana M de Araújo, Phuong T Le, Caroline de Carvalho Picoli, Rowan Hardy, Ziru Li, Francisco José A de Paula, Clifford J Rosen

**Affiliations:** Departamento de Clínica Médica, Faculdade de Medicina de Ribeirão Preto, 14049-900, Universidade de São Paulo, Ribeirão Preto, Brasil; Center for Molecular Medicine, MaineHealth Institute for Research, Scarborough, ME 04074, United States; Center for Molecular Medicine, MaineHealth Institute for Research, Scarborough, ME 04074, United States; Center for Molecular Medicine, MaineHealth Institute for Research, Scarborough, ME 04074, United States; Institute of Clinical Sciences, University of Birmingham, B15 2TT, Birmingham, United Kingdom; Center for Molecular Medicine, MaineHealth Institute for Research, Scarborough, ME 04074, United States; Departamento de Clínica Médica, Faculdade de Medicina de Ribeirão Preto, 14049-900, Universidade de São Paulo, Ribeirão Preto, Brasil; Center for Molecular Medicine, MaineHealth Institute for Research, Scarborough, ME 04074, United States

**Keywords:** nutrition, osteoporosis, corticosteroids, bone turnover, DXA, bone QCT/micro-CT

## Abstract

Caloric restriction (CR) alters energy metabolism and increases systemic glucocorticoid production, contributing to bone loss and bone marrow adipose tissue (BMAT) expansion. The enzyme 11β-hydroxysteroid dehydrogenase type 1 (11β-HSD1) mediates the local amplification of glucocorticoids within these tissues, but its contribution to CR-induced skeletal alterations remains unclear. Eight-week-old male and female WT and global 11β-HSD1 KO mice were assigned to ad libitum feeding or 30% CR for 8 wk. Body composition was assessed by DXA, bone microarchitecture by micro-CT (μCT), biomechanics by 3-point bending, BMAT by histology, serum bone turnover markers, and corticosterone by ELISA. Caloric restriction reduced body weight and lean mass of both sexes and genotypes. Fat mass was decreased in males WT and KO but not in females, while bone mass was reduced significantly in WT and KO female CR. Micro-CT revealed trabecular and cortical deterioration in both WT and KO CR females. Bone marrow adipose tissue was increased in mice under CR in both sexes, regardless of genotype. KO mice exhibited elevated corticosterone during CR, suggesting compensatory hypothalamic-pituitary-adrenal activation. These results demonstrate that CR induces sex-dependent changes in mesenchymal tissues. Under CR, females preserved fat mass but lost bone mass, while males maintained bone mass despite fat loss. Both sexes experienced lean mass loss. The effects of 11β-HSD1 deletion were also sex-specific: female KO mice had higher trabecular and cortical parameters, and lean mass under ad libitum feeding, while male KO mice under CR showed increased lean mass and trabecular bone thickness. However, 11β-HSD1 deletion did not prevent BMAT expansion in either sex under CR.

## Introduction

Caloric restriction (CR) induces metabolic, hormonal, and molecular adaptations aimed at maintaining whole-body homeostasis and constitutes a fundamental approach in the treatment of obesity. Curiously, there is evidence showing that CR increases life expectancy.^1^ There are several strategies to reduce weight such as diet combined with exercises, bariatric surgery, and more recently, the pharmacological treatment with GLP1 receptor agonists. Weight loss in people with obesity can improve insulin sensitivity, cardiovascular health, quality of life, and decrease risk of certain cancers. Il’yasova and colleagues showed for the first time, in humans, that moderate calorie restriction can reduce oxidative stress.^2^ Unfortunately, it seems that the internal environment elicited by CR is unfavorable for the skeleton. There are several clinical and experimental studies showing bone catabolism and increased fracture susceptibility induced by CR.^3–5^

Bone marrow adipose tissue (BMAT) is a metabolically active fat tissue, found within the medullary canal that plays an important role in local energy storage, as well as directly producing endocrine factors, such as leptin and adiponectin, that can influence bone metabolism.^6^ It is molecularly and metabolically distinct from other fat depots, playing a unique role in glucose homeostasis, and exerting important paracrine and endocrine functions in energy homeostasis and bone metabolism.^6^ Equivalent to roughly 10% of the body’s adipose tissue in healthy adults,^7^^,^^8^ BMAT can expand or shrink in response to an array of exogenous and endogenous stimuli. These include an expansion upon aging, chronic glucocorticoid use, chemotherapy and radiotherapy, estrogen deprivation, leptin deficiency, and CR as occurs in anorexia nervosa (AN).^9–11^ This expansion of BMAT is associated with an increased risk of osteoporosis and fracture, and may further exacerbate bone loss associated with pathological conditions.^12–15^

Importantly, sexual dimorphism in body composition and bone metabolism has received limited investigation in experimental models of CR. In humans and rodents, males and females display distinct patterns of fat distribution, muscle mass, and bone density, driven by differences in sex steroids and their interaction with metabolic pathways. In murine models, the innate tendency of expansion exhibited by BMAT in conditions of CR persists and appears to show sexual dimorphism.^16–19^ One study reported that CR induced a notable elevation in circulatory levels of the endogenous glucocorticoid in mice and hypothesized that glucocorticoids might drive the observed BMAT expansion in this condition.^17^ These changes in BMAT and circulating cortisol levels are also paralleled in women with AN.^20^

In clinical settings, there are striking differences in adipose tissue distribution and bone phenotype between primary obesity and obesity due to endogenous hypercortisolism. Primary obesity characteristically shows increased bone mass in combination with increased intrahepatic lipids (IHL), subcutaneous and visceral adipose tissue (SAT and VAT), but normal BMAT.^21^ In contrast, obesity secondary to hypercortisolism is associated with decreases in bone mass, in combination with reduced SAT, but a marked expansion of IHL, VAT, as well as BMAT.^11^ Consequently, glucocorticoids drive an unhealthy distribution of VAT, ectopic deposition of lipids, BMAT expansion, and bone loss. However, whether circulating glucocorticoids underpin and exacerbate the expansion of BMAT in response to CR, in conditions such as AN remains to be determined.

Glucocorticoids have a unique pattern of regulation, the classical hypothalamic-pituitary-adrenal (HPA) axis and accounts for a fine-tuned regulation at tissue level. The 11β-hydroxysteroid dehydrogenase type 1 (11β-HSD1) enzyme converts the inert steroid metabolite cortisone into its active endogenous counterpart cortisol. This enzyme has been shown to be highly expressed in multiple adipose depots, and in conditions of obesity, as well as directly upregulated by glucocorticoids themselves.^22^^,^^23^ To date, numerous studies in murine models have revealed that in response to exogenous glucocorticoids, changes in fat depots, such as VAT, IHL, and SAT, are dependent on the activity of 11β-HSD1, with its deletion protecting from metabolic derangements and overexpression inducing metabolic syndrome.^24–26^ Similarly, 11β-HSD1 KO animals are protected from exogenous glucocorticoid-induced osteoporosis, avoiding both, the decreased activity of osteoblasts and the apoptosis of osteoblasts and osteocytes.^27^

Based on these findings, we predict that CR drives increased systemic adrenal glucocorticoid output in combination with an upregulation of 11β-HSD1 and local glucocorticoid activation within BMAT. We hypothesize that these changes reflect a key skeletal adaptation to energy shortage, contributing to BMAT expansion and bone loss. The present study aims to investigate the contribution of 11β-HSD1 and local glucocorticoid activation toward changes in BMAT expansion and bone remodeling in response to CR in mice.

## Materials and methods

### Ethics statement

All the experiments followed the US National Research Council’s Guide for the Care and Use of Laboratory Animals and the US Public Health Service’s Policy on Humane Care and Use of Laboratory Animals. The study protocol was approved by the IACUC of the Maine Medical Institute for Research (MHIR) (IACUC #2209).

### Animals and CR protocol

Eight-week-old female and male mice with global deletions of 11β-HSD1 (KO) along with age-matched WT littermates were used. The mice used in this study were on a C57BL/6J genetic background. KO mice were generated by crossing 11β-HSD1 floxed mice with the ZP3-Cre expressing strain to achieve germline deletion of 11β-HSD1, as previously described.^28^ All mice were maintained on a 10 h light/14 h dark cycle at the animal facility at MHIR. All mice had ad libitum (AL) access to water, and body weight was monitored weekly throughout the experiment.

Animals were divided into 4 groups (*n* = 10 per group): WT and KO for AL groups, and WT and KO for CR groups. Animals were 8 wk old at protocol initiation and were maintained on their respective diets for 8 wk.

Ad libitum diet groups had unrestricted access to standard chow (AIN93M, D10012Mi, Research Diets, Inc.). Caloric restriction mice received 30% fewer calories than AL groups using a modified AIN-93M diet formulated for 30% CR (D06112301i, Research Diets, Inc.). Ad libitum diet food intake was measured weekly, while CR diet was fed daily. The CR diet-maintained protein, mineral, and vitamin levels while restricting caloric intake.

### Dual-energy X-ray absorptiometry (DXA)

All 80 mice underwent DXA exams. BMD, BMC, lean mass, and fat mass assessments were conducted using a FAXITRON UltraFocus DXA system (Tucson, AZ) at 2 time points: 8 wk (basal) and 16 wk of age.

### Micro-CT (μCT)

Right femurs were preserved in 70% ethanol and subsequently scanned using a μCT system (vivaCT 40, Scanco Medical AG). Scans were acquired with an isotropic voxel size of 10.5 μm^3^, using an X-ray tube voltage of 70 kVp, current of 114 μA, and an integration time of 250 ms. Images were processed with Gaussian filtering and segmented using standard thresholds. Trabecular bone parameters were assessed at the distal metaphysis, including bone volume fraction (BV/TV, %), trabecular thickness (Tb.Th, mm), trabecular number (Tb.N, mm^−1^), and trabecular separation (Tb.Sp, mm). Cortical bone was evaluated at the midshaft for bone area fraction (Ct.Ar/Tt.Ar, %), and cortical thickness (Ct.Th, mm). All analyses were conducted using the Scanco evaluation software (version 4.05). Acquisition and analysis of μCT data were performed in accordance with published guidelines.^29^

### Mechanical testing (3-point bending)

Femora were tested in 3-point bending using an electrical force materials testing machine (Electroforce 3230, Bose Corporation), with the load point in displacement control moving at a rate of 0.1 mm/s with force and displacement data collected at 60 Hz. Mechanical properties, including bending rigidity, apparent modulus of elasticity, ultimate moment, and apparent ultimate stress, were calculated using force–displacement data and mid-shaft geometry from μCT. Work to fracture was determined as the area under the force–displacement curve using the Riemann Sum method, and bending rigidity was derived from the linear portion of the curve.

### Tibia histology

Bones were fixed in 10% formalin for 24 h, and then decalcified in 14% EDTA solution for a minimum of 2 wk, with fresh EDTA solution replaced every 48 h. Following decalcification, bones were post-fixed for an additional 24 h in 10% formalin. Bones were subsequently embedded in paraffin and sectioned at 5 μm thickness and stained with H&E. Bone marrow adipocytes were assessed in the proximal tibial metaphysis. Histological images of the proximal tibia were acquired at 20× magnification. A square region of interest (ROI; 300 × 300 pixels) was manually delineated within the metaphyseal trabecular compartment using Fiji software (version 1.54f). The ROI was positioned immediately distal to the growth plate, as close as possible without contacting the growth plate cartilage, in order to standardize the analyzed metaphyseal region across samples. Bone marrow adipocytes within the ROI were manually counted using Fiji. For each animal, one histological section and one field of view were analyzed.

### Enzyme-linked immunosorbent assay (ELISA)

C-terminal telopeptides of type I collagen (CTX), procollagen type 1 N-terminal propeptide (P1NP) levels (IDS), and corticosterone (Invitrogen) were detected using the Mouse/Rat ELISA Kit according to the manufacturer’s instructions. The intra-assay variations were lower than 10%.

### Statistical analysis

Graphpad Prism 10 software was used to perform statistical tests. It was performed a 2-way ANOVA with Tukey’s post hoc test. The statistical threshold was set at α = .05. Data are presented as mean and SD.

## Results

### The lack of expression of 11β-HSD1 increases serum levels of corticosterone during CR

The alterations in circulatory levels of corticosterone in response to 11β-HSD1 deletion and CR were different between females and males. In females, the serum levels of corticosterone in KO CR (7732 ± 2284 pg/mL) were higher than KO AL (2866 ± 446 pg/mL) and WT CR (2285 ± 394 pg/mL), *p* < .05. The serum levels of corticosterone in WT AL (2059 ± 477 pg/mL) were similar between the groups (Figure S1A and S2). In males, both WT CR (2765 ± 95 pg/mL) and KO CR (4031 ± 679 pg/mL) showed higher serum levels of corticosterone in comparison to their counterparts (WT AL = 1000 ± 122; KO AL = 1805 ± 176 pg/mL), *p* < .05 (Figure S1B).

### Deletion of 11β-HSD1 does not affect body composition changes induced by CR

**Figure 1:**
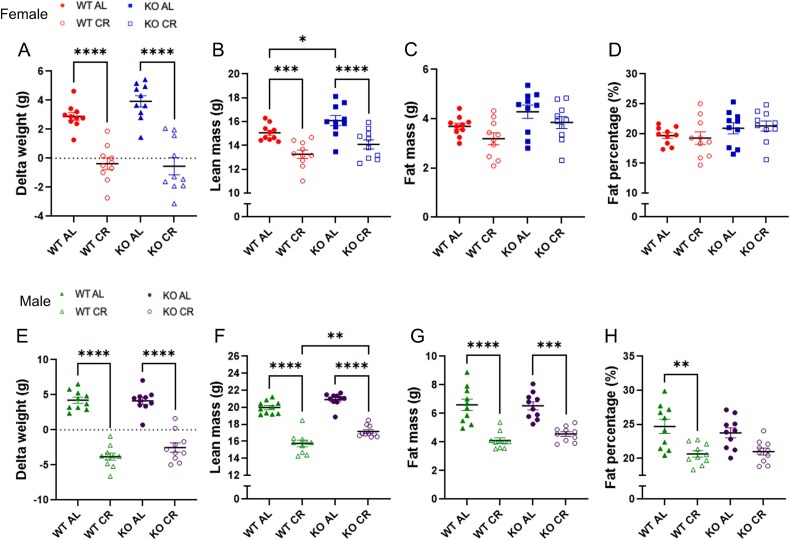
(A) Delta weight in female. (B) Lean body mass in female. (C) Fat body mass in female. (D) Fat mass percentage in female. (E) Delta weight in male. (F) Lean body mass in male. (G) Fat body mass in male. (H) Fat mass percentage in male. Lean, fat mass, and fat percentage accessed by DXA. WT AL, WT ad libitum diet; WT CR, WT caloric restriction; KO AL, KO ad libitum diet; KO CR, KO caloric restriction. Bars represent mean values and error bars indicate SD. ^*^p < .05, ^**^p < .01, ^***^p < .001, and ^****^p < .0001, two-way ANOVA with Tukey’s post hoc test. *n* = 10 per group.

After 8 wk of CR, body weight was reduced in both females and males CR groups. However, males under CR exhibited more pronounced body weight loss compared to females (Figure 1A and E).

Caloric restriction led to lean mass loss in both female and male mice. Female KO AL mice showed higher lean mass than WT AL female mice (Figure 1B). In addition, male KO mice under CR had higher lean mass than WT CR (*p* < .05) (Figure 1F). All animals under CR, regardless of sex, experienced muscle loss over time, approximately 3% in WT and KO females, 17% in WT males, and 13% in KO males.

Furthermore, all female groups did not show significant reduction in fat mass, whereas WT and KO male mice under CR exhibited a significant decrease in fat mass, *p* < .05 (Figure 1C, D, G, and H).

### Sexual dimorphism in the skeletal response to CR

**Figure 2:**
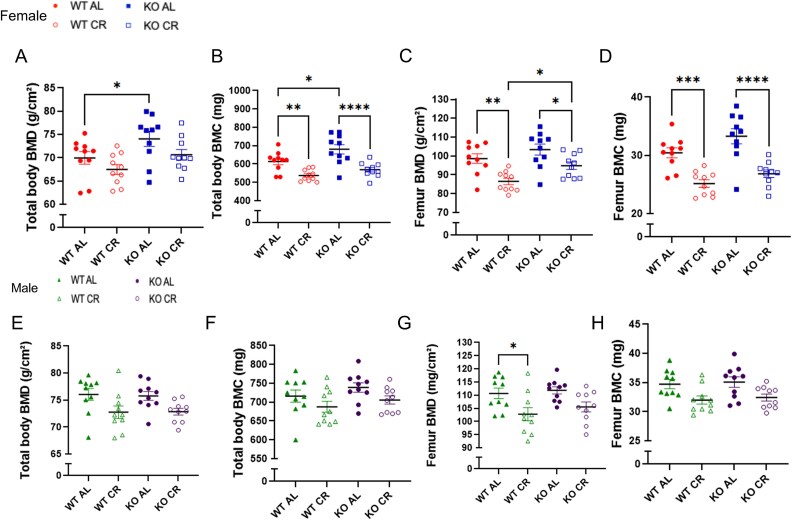
(A) Total body BMD in female. (B) Total body BMC in female. (C) Femur BMD in female. (D) Femur BMC in female. (E) Total body BMD in male. (F) Total body BMC in male. (G) Femur BMD in male. (H) Femur BMC in male. WT AL, WT ad libitum diet; WT CR, WT caloric restriction; KO AL, KO ad libitum diet; KO CR, KO caloric restriction. Bars represent mean values and error bars indicate SD. ^*^p < .05, ^**^p < .01, ^***^p < .001, and ^****^p < .0001. Two-way ANOVA with Tukey’s post hoc test. *n* = 10 per group.

Total body BMD and BMC were higher in KO females compared to WT females. Females subjected to CR of both genotypes showed lower femur BMD, BMC, and total body BMC in comparison to AL diet groups (Figure 2A-D). KO males showed skeletal protection under conditions of CR; they did not show bone loss as occurred in femur of WT CR mice. In contrast, only WT male mice exhibited lower femoral BMD under conditions of CR (Figure 2E-G).

### CR causes cortical loss in KO female mice

**Figure 3:**
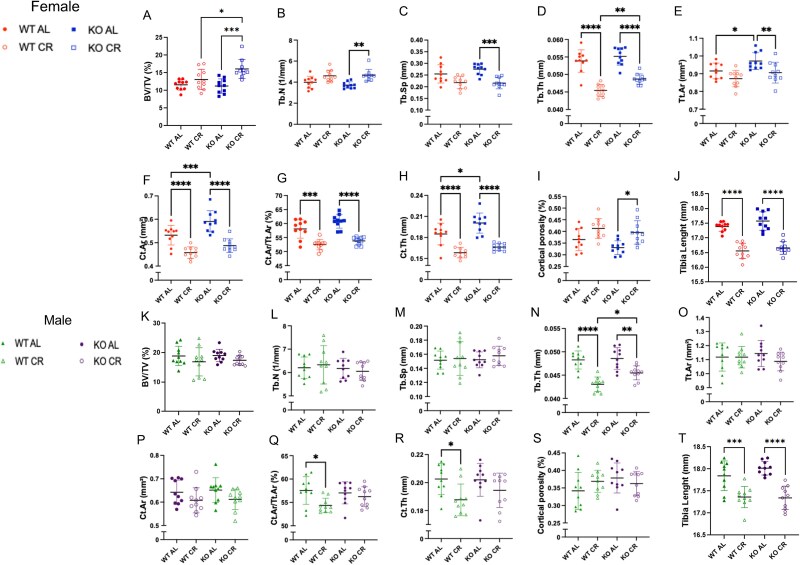
Micro-CT results. Females (A-J) and males (K-O). Trabecular bone volume (BV/TV), trabecular number (Tb.N), trabecular separation (Tb.Sp), trabecular thickness (Tb.Th), total tissue area (Tt.Ar), cortical area (Ct.Ar), and fractional cortical area (Ct.Ar/Tt.Ar). WT AL, WT ad libitum diet; WT CR, WT caloric restriction; KO AL, KO ad libitum diet; KO CR, KO caloric restriction. Bars represent mean values and error bars indicate SD. ^*^p < .05, ^**^p < .01, ^***^p < .001, and ^****^p < .0001. Two-way ANOVA with Tukey’s post hoc test. *n* = 10 per group.

In females, the BV/TV values were higher in KO CR than KO AL and WT CR (Figure 3A). In females KO, Tb.N was higher in CR compared to the KO AL, while Tb.Sp was lower in KO CR than KO AL. Additionally, Tb.Th was lower in both CR groups compared to their AL diet counterparts and higher in KO CR than in WT CR. Total area (Tt.Ar) was lower in KO CR than KO AL, and higher in KO AL than WT AL, in females (Figure 3B-E). Ct.Ar was lower in CR females compared to their respective AL diet counterpart. Furthermore, KO AL mice exhibited higher Ct.Ar than WT AL mice. Caloric restriction diet resulted in lower Ct.Ar/Tt.Ar in both WT and KO female mice. Ct.Th was reduced in the CR groups compared to their respective controls. Moreover, KO mice under AL feeding exhibited greater Ct.Th than WT AL. Conversely, KO CR females showed higher cortical porosity than KO AL. Tibia length was lower in both CR groups than their AL counterparts (Figure 3F-J).

In WT females, considering the biomechanical testing results, the ultimate moment was lower in CR than in AL, whereas in KO mice, the ultimate moment did not differ between CR and AL. Apparent bending modulus and ultimate stress were similar across all groups. It suggested that differences in bending rigidity and ultimate moment were driven by variations in bone geometry rather than changes in bone material properties. Nevertheless, CR bone material exhibited reduced energy absorption capacity at the ultimate moment compared with AL bone material (Figure S2A-D).

In males, BV/TV, Tb.N, Tb.Sp, and Tt.Ar were similar between groups (Figure 3K-O). However, Tb.Th was lower in CR groups, with KO CR showing higher Tb.Th than WT CR (Figure 3N). No differences were observed between Ct.Ar and cortical porosity in male groups (Figure 3P and S). Ct.Ar and Ct.Th were lower in WT CR compared to WT AL (Figure 3Q and R). Tibia length was lower in all groups submitted to CR (Figure 3T). The mechanical properties of the bones, the KO bones had greater bending rigidity and ultimate moment than the WT; however, there were no significant effects of CR on the bone mechanical properties or significant interaction of CR and KO, in males (Figure S2E-H).

### Bone remodeling markers are suppressed in CR males

**Figure 4:**
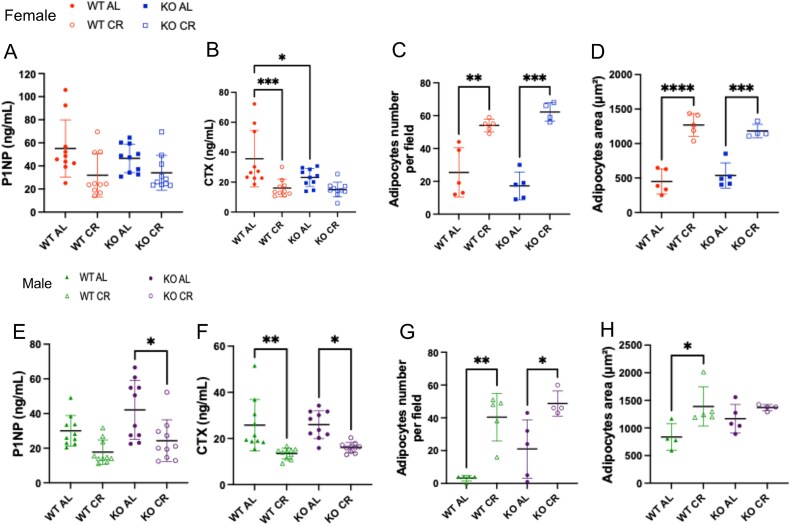
(A) Circulatory procollagen type 1 N-terminal propeptide (P1NP) in female. (B) Circulatory C-terminal telopeptide of type I collagen (CTX) in female. (C) Bone marrow adipocyte number per field in female. (D) Bone marrow adipocytes area in female. (E) Circulatory P1NP in male. (F) Circulatory CTX in male. (G) Bone marrow adipocyte number per field in male. (H) Bone marrow adipocytes area in male. Bone marrow adipocyte quantification in the proximal tibial metaphysis. Images were acquired at 20× magnification and analyzed using a standardized 300 × 300 pixels ROI positioned immediately distal to the growth plate. Bone marrow adipocytes were manually counted using ImageJ Fiji (version 1.54f). One section and one field per animal were analyzed. Each dot represents one animal. WT AL (*n* = 5), WT CR (*n* = 5), KO AL (*n* = 5), and KO CR (*n* = 4). WT AL, WT ad libitum diet; WT CR, WT caloric restriction; KO AL, KO ad libitum diet; KO CR, KO caloric restriction. Bars represent mean values and error bars indicate SD. ^*^p < .05, ^**^p < .01, ^***^p < .001, and ^****^p < .0001, two-way ANOVA with Tukey’s post hoc test.

Serum levels of P1NP were similar between female groups (WT AL: 55 ± 25; WT CR: 32 ± 19; KO AL: 46 ± 12; KO CR: 34 ± 15 ng/mL). CTX was lower in WT CR and KO AL compared to WT AL females (WT AL: 36 ± 19; WT CR: 16 ± 6; KO AL: 23 ± 6; KO CR: 15 ± 5 ng/mL), which may partially contribute to the higher bone mass in AL KO mice (Figure 4A and B). In males, P1NP levels were lower in KO CR compared to KO AL (WT AL: 30 ± 9; WT CR: 18 ± 7; KO AL: 42 ± 17; KO CR: 24 ± 12 ng/mL, *p* < .05). In addition, males presented CTX levels lower in both CR groups compared to their respective AL counterparts (WT AL: 26 ± 11; WT CR: 13 ± 2; KO AL: 26 ± 6; KO CR: 16 ± 2 ng/mL, *p* < .05) (Figure 4E and F).

### 1‌1β-HSD1 deletion does not affect BMAT accumulation during CR

**Figure 5:**
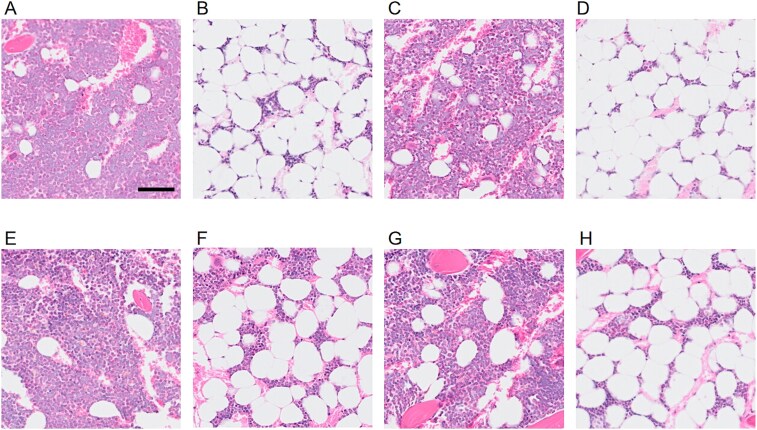
Representative HE images of bone marrow adipose tissue in tibia. (A) WT ad libitum diet (AL) female. (B) WT caloric restriction (CR) female. KO AL female. (D) KO CR female. (E) WT AL male. (F) WT CR male. (G) KO AL male. (H) KO CR male. Image acquired at 20× magnification. Scale bar: 100 μm.

In females, both adipocyte number and area were higher in CR groups compared to their respective AL counterparts (Figure 4C and D; Figure S5A-D). In males, adipocyte number was higher in both CR groups, while adipocyte area was higher only in WT CR compared to WT AL, but not in KO CR in comparison to WT CR (Figure 4G and H). Figure 5 shows representative HE images of BMAT in tibia of males and females, both WT and KO.

## Discussion

While CR is widely employed as a therapeutic strategy in the clinical management of several diseases, it poses a physiological challenge that induces a range of metabolic and hormonal adaptations, including an increase in glucocorticoid production.^17^^,^^30^ This condition promotes weight loss, often accompanied by variable degrees of fat and lean mass depletion. The skeletal consequences are particularly distinctive, as CR leads to bone loss while simultaneously promoting BMAT expansion. A growing body of evidence suggests that glucocorticoids play a central role in mediating these CR-induced skeletal alterations. In this context, we conducted an 8-wk dietary intervention involving 30% CR to investigate whether genetic deletion of 11β-HSD1 affects body composition, BMAT accumulation, and bone mass in both male and female mice.

In the present study, all animals under CR exhibited a progressive loss of muscle mass over time, regardless of sex. In the study by Picoli et al., animals treated with semaglutide exhibited a reduction in food intake and lean mass loss during the treatment period,^31^ supporting the hypothesis that energy restriction per se is sufficient to reduce lean mass. Furthermore, under AL feeding, female KO mice exhibited higher lean mass than their WT counterparts, and a similar pattern was observed in males under CR, with KO CR animals showing greater lean mass than WT CR mice. A previous study showed that myostatin, a negative regulator of muscle growth, is involved in muscle loss under hypercortisolemic conditions, and that 11β-HSD1 KO animals exhibit lower myostatin expression.^26^ In addition, myostatin gene deletion blocks glucocorticoid-induced upregulation of atrogenes and proteasomal activity.^32^ Although myostatin expression was not assessed in the present study, variation in this peptide may contribute to differences in lean mass loss or preservation observed among the experimental groups.

Analysis of fat mass and bone mass findings revealed a sexual dimorphism. Males exhibited fat loss while maintaining bone mass, whereas females preserved fat mass but experienced bone mass loss. Similar sex-specific patterns have been reported in other models; for example, male mice lacking the vitamin D receptor displayed reduced fat mass without bone loss, whereas females exhibited bone impairment without changes in fat mass.^33^ Previous investigations have demonstrated that females under CR, in comparison to males, exhibit lower intensity of lipolysis, energy expenditure, and fatty acid oxidation, alongside increased postprandial lipogenesis, which may underlie the preservation of fat mass under these conditions.^18^ Estrogen is implicated in mediating this female-specific resistance to fat loss during CR, as females appear resistant to adipocyte hypotrophy and lipolysis, in addition to potential suppression of energy expenditure.^34^ Moreover, weight loss leads to reduced mechanical loading on the skeleton, which likely contributes to the observed bone mass loss.^35^

During CR, the body undergoes physiological adaptations to reduce energy expenditure, providing a survival advantage but potentially causing deleterious effects in the long term. In AN, for example, in addition to adaptations in energy metabolism, bone formation is suppressed while bone resorption is increased, as demonstrated in both animal and human studies.^36^^,^^37^ In the present study, both groups of females under CR exhibited reduced bone mass. Micro-CT analysis revealed that CR females showed decreased values of cortical and trabecular parameters, and greater cortical porosity. However, bone deterioration was attenuated in KO CR females, in view that KO CR showed significant increase in femur BMD, BV/TV, and Tb.Th compared to WT CR. Conversely, males under CR did not exhibit decreased bone mass by DXA, only decreased femur BMD in WT CR. In addition, in males, μCT analysis detected impairment only in Tb.Th and tibia length in both CR groups. In addition, results showed lower cortical parameters in WT CR. Males exhibited a milder impact on bone. The results are similar to previous study that the authors observed that mice under CR showed preserved trabecular bone in males under CR-feeding.^38^

The data suggest that deletion of 11β-HSD1 modulates bone turnover in a sex- and diet-dependent manner. In females, reduced bone resorption under AL feeding may contribute to the higher bone mass observed in KO mice, whereas CR appears to blunt the expected activation of bone remodeling. In males, the reduction in both resorption and formation markers under CR indicates a generalized suppression of bone turnover, which may help preserve bone mass in the short term but potentially at the cost of impaired skeletal renewal. Together, these findings support the concept that maintenance of bone mass during CR, particularly in males, may rely on reduced bone remodeling rather than active bone formation.

Our data suggest that deletion of 11β-HSD1 did not protect animals from the CR-induced increase in bone marrow adipocyte number in either sex. These findings are partially consistent with a previous study, which reported increased BMAT in females but a protective effect in KO males under CR regarding BMAT expansion.^19^ That study concluded that corticosterone plays only a minor role in BMAT accumulation, whereas progesterone appears to be more strongly associated with this effect.^19^ There are methodological differences between the present study and that of Lovdel et al. In our model, CR was initiated at 8 wk of age and maintained for 8 wk, whereas Lovdel et al. started CR at 9 wk of age and evaluated animals after 6 wk of intervention. Differences in the duration of CR may have influenced the endocrine adaptations to CR. Similarly to Lovdel et al., Schill et al. showed that corticosterone does not significantly contribute to BMAT expansion. The authors proposed that glucocorticoids may primarily affect early stages of adipocyte differentiation within the bone marrow, while having minimal influence on mature marrow adipocytes.^39^

In the present study, BMAT expansion in KO mice was comparable to WT animals under CR in both sexes, despite elevated serum corticosterone levels. One possible hypothesis is that this effect reflects insufficient inactivation of corticosterone by 11β-HSD2, an enzyme that is expressed at low levels in the bone marrow; however, this mechanism was not directly tested in the present study.^19^ Elevated corticosterone levels may result from a compensatory mechanism, the overactivation of the HPA axis and increased glucocorticoid receptor expression.^40^^,^^41^ These findings suggests that deletion of 11β-HSD1 is insufficient to prevent CR-induced expansion of BMAT in the context of elevated systemic glucocorticoid levels.

In summary, CR induces complex, sex-dependent alterations in mesenchymal tissues. Females preserve fat mass but lose bone, whereas males lose fat mass while largely preserving bone. Both sexes exhibit loss of lean mass under CR. The impact of 11β-HSD1 deletion differs between sexes, with female KO mice displaying greater BMC, BMD, and lean mass under AL-feeding and reduced CTX levels, and male KO mice showing partial preservation of muscle mass and trabecular thickness under CR.

### Limitations

We did not assess free corticosterone levels or local corticosterone concentrations within the bone marrow microenvironment, which would be more directly related to marrow adipocyte differentiation and osteoblast–osteoclast activity. Future studies incorporating measurements of free corticosterone and bone marrow glucocorticoid content will be necessary to clarify the contribution of local glucocorticoid metabolism to CR-induced skeletal and BMAT alterations.

## Supplementary Material

Supplemental_File_ziag075

## Data Availability

Data will be made available upon reasonable request.
