# Supplementary material for: 11β-Hydroxysteroid dehydrogenase type 1 deficiency causes sexual dimorphism in body composition and bone mass in response to caloric restriction
Source: JBMR Plus. 2026 Apr 17;10(6):ziag075. doi: 10.1093/jbmrpl/ziag075 (PMC13171033; doi:10.1093/jbmrpl/ziag075)
Supplement: Supplemental_File_ziag075 [file supplemental_file_ziag075.docx]

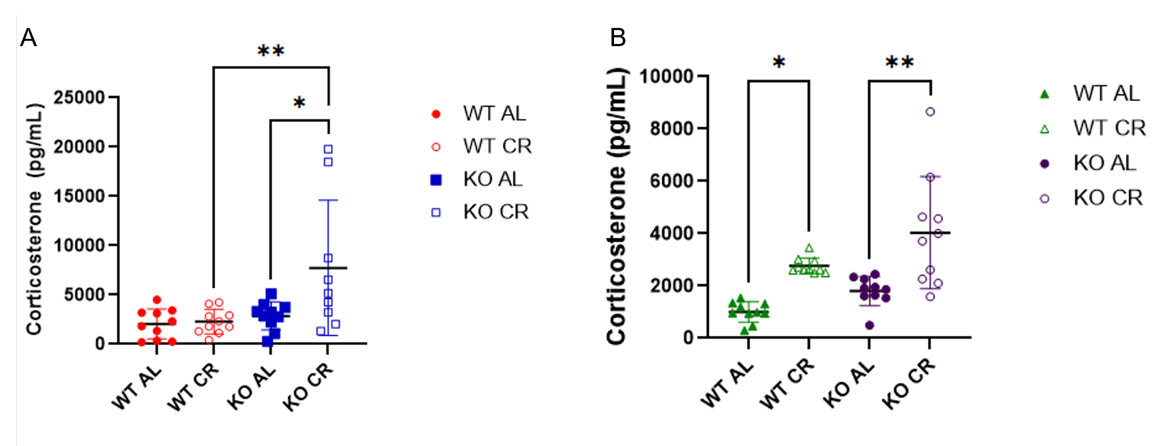


Supplemental Figure 1. A – Circulatory corticosterone in female mice. B – Circulatory corticosterone in male mice. WT AL = wild type a*d libitum* diet, WT CR = wild type caloric restriction, KO AL = knockout a*d libitum* diet, and KO CR = knockout caloric restriction. n = 10 per group


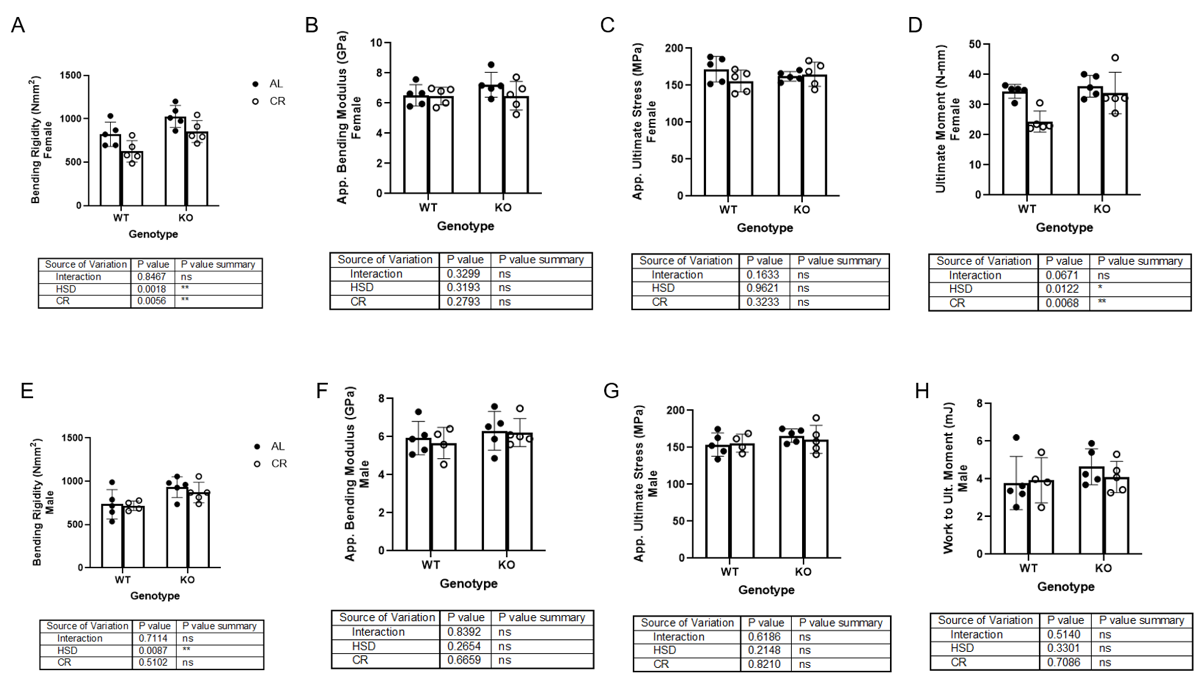


Supplemental Figure 2. Biomechanical testing results. A – Bending rigidity in female mice. B – Apparent bending modulos in female mice. C – Apparent ultimate stress in female mice. D – Ultimate moment in female mice. E – Bending rigidity in male mice. F – Apparent bending modulos in male mice. G – Apparent ultimate stress in male mice. F – Ultimate moment in male mice. WT AL = wild type a*d libitum* diet, WT CR = wild type caloric restriction, KO AL = knockout a*d libitum* diet, and KO CR = knockout caloric restriction. n = 5 per group
